# Subacute Thyroiditis Causing Fever of Unknown Origin: A Reminder of the Uncommon Cause

**DOI:** 10.7759/cureus.47037

**Published:** 2023-10-14

**Authors:** Sin Yin Lee, Chaozer Er

**Affiliations:** 1 General Medicine, National University of Singapore, Singapore, SGP; 2 General Medicine, Woodlands Health, Singapore, SGP

**Keywords:** viral thyroiditis, puo ( pyrexia of unknown origin), syndrome of fever of unknown origin, subacute thyroiditis, fever of unknown origin

## Abstract

Subacute thyroiditis (SAT) is a transient inflammation of the thyroid gland that often occurs following a viral infection. It is an infrequent cause of fever of unknown origin (FUO). We present a 46-year-old gentleman who presented with two weeks of fever and some non-specific left-sided neck pain. His initial investigations and microbiological workup were unremarkable. He did not report any hyperthyroid symptoms. A computed tomography of the neck, chest, abdomen, and pelvis showed a heterogeneous appearance of his thyroid gland. Thyroid function was then performed, and it showed primary hyperthyroidism. His thyroid autoantibodies were negative. Ultrasonography of his thyroid showed features consistent with thyroiditis. He was treated with a course of oral steroids. His fever lysed. His thyroid function turned from a primary hyperthyroid pattern to subclinical hypothyroidism. His anti-thyroglobulin antibody level remained elevated after the steroid treatment. Our case highlights that SAT is an uncommon cause of FUO in patients without specific localizing symptoms. It can present without overt hyperthyroid clinical features. Steroid treatment is useful. There may be value in monitoring the anti-thyroid antibodies in SAT’s management.

## Introduction

Fever of unknown origin (FUO) was defined as a temperature exceeding 38.3°C on at least three occasions over at least three weeks, with no diagnosis made despite one week of inpatient investigation [1]. With medical advancements, the minimum diagnostic evaluation of FUO has been revised to be three outpatient visits or three days of in-hospital investigation [1]. The etiologies of FUO can be grouped into four main categories, namely non-infectious inflammatory disorders, infections, malignancies, and miscellaneous [2]. Subacute thyroiditis (SAT) is a self-limiting inflammation of the thyroid gland, usually triggered by a previous viral infection, and is considered as an infrequent miscellaneous cause of FUO. It is often overlooked, as typical symptoms may be absent [3]. We hereby present a case of FUO caused by SAT without typical symptoms of hyperthyroidism and significant neck pain.

## Case presentation

A 46-year-old gentleman with no significant past medical history was referred to our hospital by the primary care physician due to a two-week history of intermittent fever. Two weeks prior to this hospitalization, he experienced fever, headache, and cough for three days. Computed tomography (CT) of the brain showed bilateral maxillary, ethmoid, and right frontal sinusitis. He was admitted for one day and treated for sinusitis with a course of oral co-amoxiclav.

Five days post-discharge, he visited the primary care due to a persistent fever. He reported a left-sided headache and non-specific discomfort at the left sternocleidomastoid muscle area. He is a non-smoker and does not drink alcohol or use any illicit substances. The systematic review and physical examination were unremarkable. Laboratory results showed deranged liver enzymes (Table 1).

**Table 1:** Relevant investigations. Ab, antibody; AFB, acid-fast bacillus; Ag, antigen; ALP, alkaline phosphatase; ALT, alanine transaminase; ANA, antinuclear antibodies; AST, aspartate transaminase; CMV, cytomegalovirus; CRP, C-reactive protein; CT, computed tomography; CXR, chest X-ray; EBV, Epstein-Barr virus; ESR, erythrocyte sedimentation rate; GGT, gamma-glutamyl transferase; HBS, hepatobiliary system; Ig, immunoglobulin; PCR, polymerase chain reaction; TAP, thorax, abdomen, pelvis; TB, tuberculosis; TPO, thyroid peroxidase; TRAb, thyroid-stimulating hormone receptor antibody; TSH, thyroid-stimulating hormone; US, ultrasound.

Investigation	Result	Reference range
Hemoglobin	11.7 (low)	13.1-17.4 g/dL
WBC	9.20	3.82-9.91 x 10^9^/L
Platelets	392	173-414 x 10^9^/L
Ferritin	1460 (high)	30-400 ng/mL
Iron saturation	16	15-50%
CRP	25.8 (high)	1-5 mg/L
ESR	110 (high)	1-16 mm/h
Albumin	40	35-50 g/L
Total bilirubin	8	3-21 mmol/L
ALT	320 (high)	10-44 U/L
AST	123 (high)	10-21 U/L
ALP	269 (high)	25-122 U/L
GGT	246 (high)	11-50 U/L
Hepatitis A Ab, IgM	Non-reactive	
Hepatitis B surface antigen	Non-reactive	
Hepatitis B core Ab	Non-reactive	
Hepatitis C Ab	Non-reactive	
HIV Ag-Ab screen	Non-reactive	
Dengue NS1 Ag	Negative	
Dengue IgG	Negative	
Dengue IgM	Negative	
CMV IgM	Negative	
CMV IgG	Reactive: 221.0 AU/mL	
Anti-EBV IgM	Negative	
Anti-EBV IgM	Negative	
Malaria parasite screen	Negative	
Blood culture	Negative	
Urine culture	Negative	
Sputum AFB smear	Negative	
Sputum TB PCR	Negative	
Malaria parasite screen	Negative	
ANA	Negative	
Free thyroxine	65.7 (high)	12-22 pmol/L
TSH	0.01 (low)	0.27-4.20 mIU/L
Free T3	10.5 (high)	2.4-6.0 pmol/L
TPO Ab	15	5-34 IU/mL
TRAb	1.0	0.0-1.7 IU/L
US HBS	No hepatic lesion or biliary ductal dilatation is seen	
	Diameter of the common bile duct measures 0.3 cm	
CXR	No consolidation	
CT neck	Some heterogeneous appearance of thyroid gland.	
CT TAP	No foci of infection, no lymphadenopathy, no malignancy	
US thyroid	Mildly enlarged thyroid with diffusely heterogeneous echogenicity which may represent thyroiditis	

**Figure 1:**
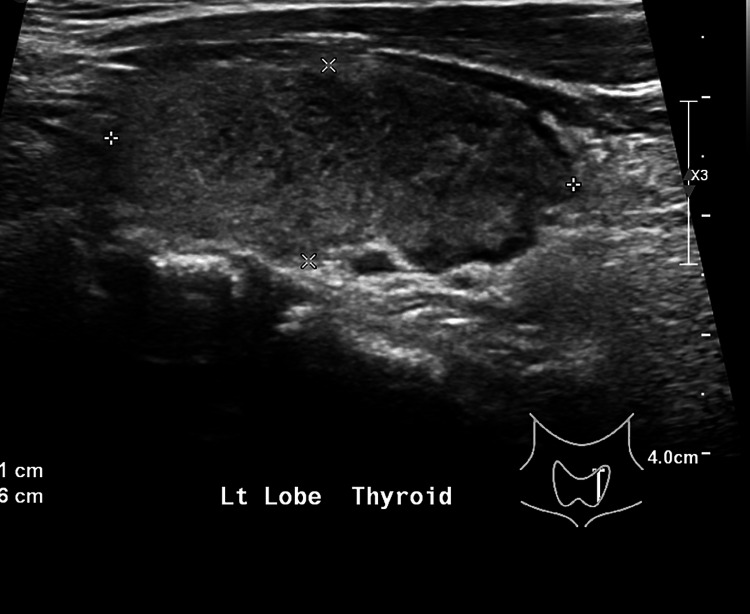
Thyroid US showed a mildly enlarged thyroid (left lobe) with diffusely heterogeneous echogenicity, suggestive of thyroiditis. US: ultrasonography.

**Figure 2:**
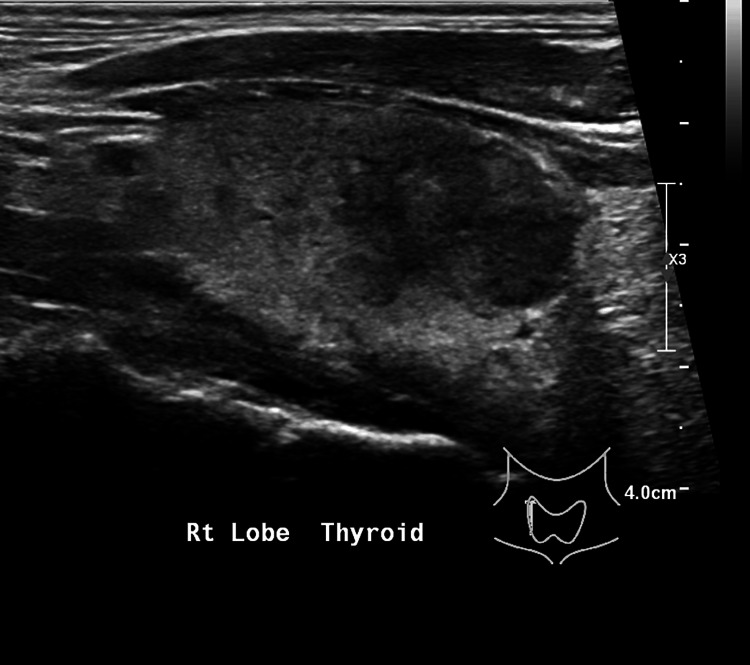
Similar features were seen on thyroid US of the right lobe. US: ultrasonography.

Subsequent investigation showed a markedly elevated erythrocyte sedimentation rate (ESR). His microbiological workup, including urinalysis, urine culture, blood culture, viral hepatitis A, B, and C, malaria microscopy, dengue antigen and antibody, COVID-19 antigen, Epstein-Barr virus antibody, and cytomegalovirus antibody (Table 1), were unremarkable. Ultrasonography (US) of the liver showed no hepatic lesions or biliary dilatation. On CT of the neck, some heterogeneous appearance of the thyroid gland was picked up as an incidental finding. Following that, a thyroid function test (TFT) was done, showing primary hyperthyroidism. However, thyroid-stimulating hormone receptor antibody (TRAb) and anti-thyroid peroxidase (TPO) antibody were negative. Thyroid US showed a mildly enlarged thyroid with diffusely heterogeneous echogenicity (Figures 1, 2), suggestive of thyroiditis (see Table 1 for investigation results).

Our endocrinologist opined that the overall clinical picture was consistent with SAT, possibly precipitated by the recent viral sinusitis. His deranged liver enzymes were attributed to hyperthyroidism. The patient was treated with propranolol 20 mg thrice daily and prednisolone 30 mg once daily (OD). The fever subsequently lysed, and he improved clinically. After three weeks of treatment, his TFT turned from primary hyperthyroidism to subclinical hypothyroidism pattern. Prednisolone and propranolol were discontinued. One month later, his TFT remained as a subclinical hypothyroid picture, but his anti-thyroglobulin antibody (TgAb) was found to be markedly elevated, raising the concern of permanent hypothyroidism. As he had an upcoming elective surgery, thyroxine replacement of 25 mg OD was initiated. A month later, his TFT was repeated as part of preoperative workup. Results continued to show subclinical hypothyroidism (see Table 2 for TFT trending). The surgery was uneventful, and his liver enzymes normalized one month after discharge.

**Table 2:** TFT trend. NR: normal range; TFT: thyroid function test; TSH: thyroid-stimulating hormone; TgAb: thyroglobulin antibody. *The reference range of laboratory values of the healthcare institution that the patient visited after discharge. He was admitted to one hospital but followed up at another hospital. The reference ranges were therefore slightly different.

Timeline	Free thyroxine (NR 12-22 pmol/L)	TSH (NR 0.27-4.20 mIU/L)	Anti-TgAb (NR 0-100 IU/mL)
On admission	65.7	0.01	NT
On discharge	43.5	0.007	NT
	Free thyroxine (NR 8-16 pmol/L)*	TSH (NR 0.45-4.50 mIU/L)*	Anti-TgAb (NR 0-100 IU/mL)*
Firstfollow-up (three weeks post-discharge)	9	8.28	NT
Second follow-up (seven weeks post-discharge, thyroxine replacement started during this clinic visit)	8	6.73	128
Preoperative (11 weeks post-discharge)	15.4	7.00	NT

## Discussion

Endocrine causes of FUO include thyrotoxicosis, adrenal insufficiency, pheochromocytoma, and SAT [3]. Among these, SAT may be overlooked as the typical neck pain and hyperthyroidism symptoms may be absent [3]. Studies have shown that the clinical characteristics of SAT are changing in recent years, with increasingly more cases of painless SAT [4]. Stasiak et al. postulated that this may result from a changing disease course or that previously painless SAT was simply undiagnosed due to poorer diagnostic possibilities [4]. SAT affects women more often than men [5].

Our case was a male patient who had a history of a recent preceding viral infection but had no obvious neck pain or tenderness and lacked symptoms of thyrotoxicosis. Given the potential for SAT to be painless, our case demonstrates the importance of history-taking to elicit pertinent medical history of preceding viral illnesses. Upon excluding infections, malignancies, autoimmune, and rheumatic causes, it is crucial to consider SAT as one of the diagnoses, even in a male patient. It also suggests the utility of including a TFT in routine preliminary investigations for FUO.

The case supports the role of anti-thyroid antibodies in prognosticating permanent hypothyroidism as a complication of SAT. The minimal role of autoimmunity used to be suggested, as SAT is thought to be part of a post-viral inflammatory process [6]. However, recent literature has challenged this perspective. The proposed pathogenesis of permanent hypothyroidism in SAT is that of autoimmunity and the resultant production of anti-thyroid antibodies by autoreactive B cells may have been triggered by the inflammation [7].

In one study comparing the prevalence of anti-thyroid antibodies in patients with SAT to those with painful autoimmune thyroiditis, it was found that titers of positive anti-TgAb decreased or disappeared within four months to six years after the onset of SAT [6]. This temporal elevation in anti-TgAb differs greatly from painful autoimmune thyroiditis, which shows persistently high values of anti-thyroid antibodies [6].

Our case, which showed subclinical hypothyroidism with raised anti-TgAb, considers the possibility that a higher or persistent anti-TgAb titer in SAT might be associated with a higher chance of developing persistent hypothyroidism and might require long-term levothyroxine supplementation. Positive anti-TPO was also observed to be a risk factor for permanent hypothyroidism in one study [8]. Serial measurement and monitoring of anti-thyroid antibodies during follow-up of SAT patients may help in predicting its disease course.

SAT can be treated with glucocorticoids or non-steroidal anti-inflammatory drugs (NSAIDs) [9]. Glucocorticoid shortens disease duration and achieves early clinical remission compared to NSAIDs [8]. Its effect on long-term hypothyroidism remains unclear. One study showed significantly more patients treated with corticosteroid had a diagnosis of hypothyroidism, but another study showed steroid therapy had a protective effect against permanent hypothyroidism [8,10].

## Conclusions

SAT is a rare but possible cause of FUO. We suggest TFT as a routine evaluation for FUO. Glucocorticoid treatment achieves early remission, and all patients need to be followed up for potential hypothyroidism, especially those with high anti-thyroid antibodies. 
